# Phytochemical Screening and Antidiabetic, Antihyperlipidemic, and Antioxidant Effects of *Leptopus Cordifolius* Decne. In Diabetic Mice

**DOI:** 10.3389/fphar.2021.643242

**Published:** 2021-04-08

**Authors:** Shahid Rahman, Gul Jan, Farzana Gul Jan, Hafeez Ur Rahim

**Affiliations:** ^1^ Pharmacology Lab, Department of Botany, Abdul Wali Khan University Mardan, Khyber Pakhtunkhwa, Pakistan; ^2^ Key Laboratory of Industrial Ecology and Environmental Engineering (Ministry of Education), School of Environmental Science and Technology, Dalian University of Technology, Dalian, China

**Keywords:** antidiabetic, alloxan, lipid profile, antioxidant., leptopus cordifolius

## Abstract

Plants are well known in traditional herbal medicines for their hypoglycemic and hypolipidemic activities and are often used due to their accessibility, affordability, and corollary effects. *Leptopus cordifolius* has been reported to control diabetes in folkloric medicine, but no known scientific research has been conducted to assess the plausibility of this assertion. Therefore, the current study is aimed to investigate the antidiabetic and hypolipidemic effects of *Leptopus cordifolius* leaves in alloxan-induced diabetic mice. The antidiabetic and antihyperlipidemic evaluation was conducted in Swiss albino mice at doses of 150–250°mg/kg for 15°days. The blood glucose, total cholesterol, triglyceride, LDL, HDL, creatinine, ALP, SGPT, and SGOT levels were estimated according to standard procedures. Phytochemicals of leaves were analyzed using GC-MS analysis. Enzymatic antioxidant activity of the plant was investigated spectrophotometrically by carrying out superoxide dismutase, peroxidase, and catalase assays. The membrane stabilization potential of *L. cordifolius* leaf extracts was carried out using an *in vitro* haemolytic assay. The results revealed a dose response effect with the methanolic extract of *L. cordifolius* which had significant antihyperglycemic effects at 150–250°mg/kg in alloxan treated mice, although less than the positive control (glibenclamide). Hyperlipidemic activity was significant at 250 mg/kg. The biochemical parameters, such as total cholesterol, triglyceride, LDL, HDL, creatinine, ALP, SGPT, and SGOT, were significantly improved (*p* < 0.01) by the methanolic extract of 250 mg/kg compared to the diabetic group. Treatment for 15 days showed significant elevation (*p* < 0.01) of antioxidant enzymes. GC-MS analysis provided tentative identifications of 52 compounds in the methanolic extract of *L. cordifolius,* of which 12 compounds have reported antidiabetic activity. In conclusion, methanolic extract *of L. cordifolius* of 150 and 250°mg/kg body weight showed significant antidiabetic and antihyperlipidemic activities in alloxan-induced diabetic mice and, with further work, has the potential to be used to manage blood glucose and cholesterol levels.

**Figure:**
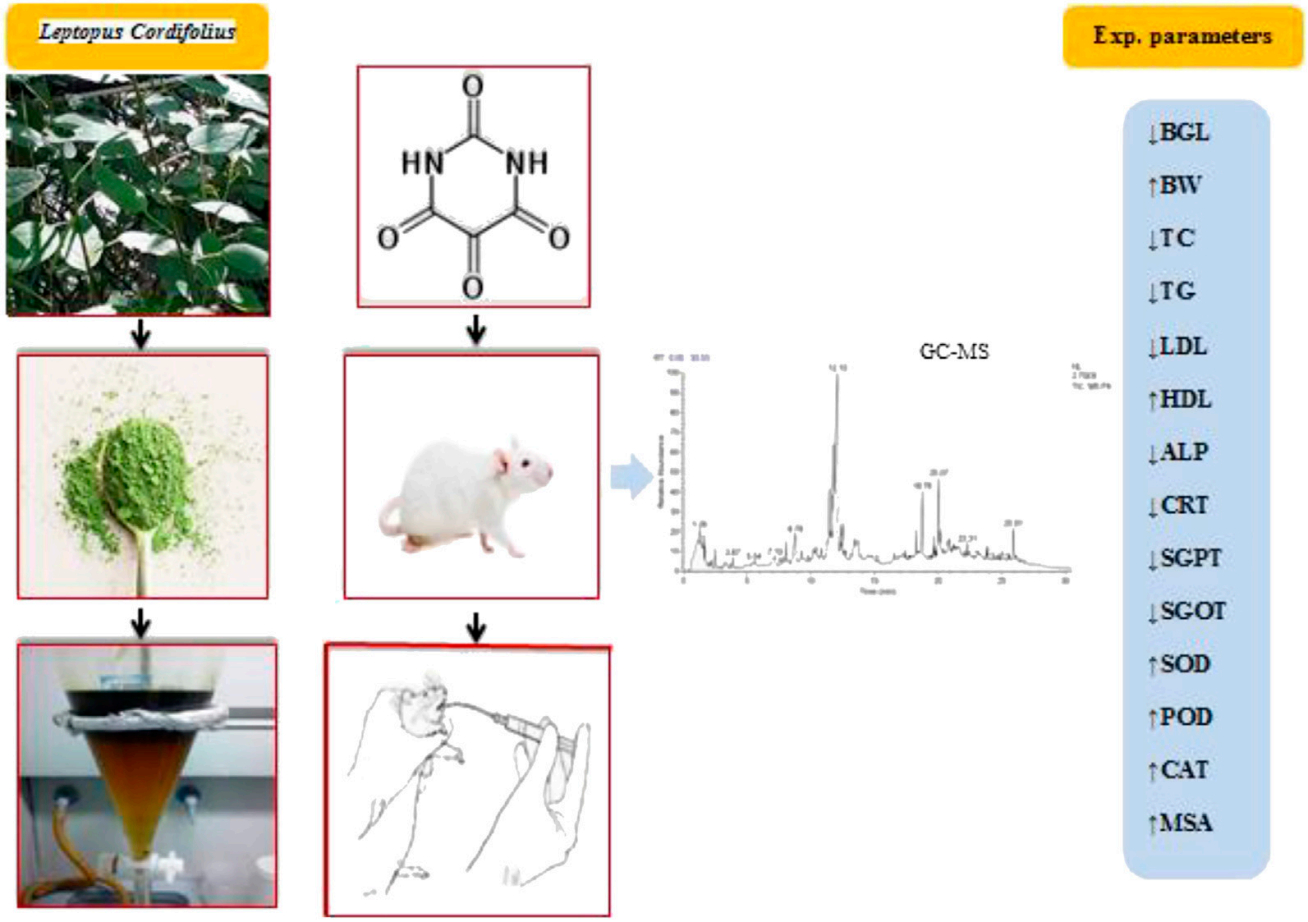
**GRAPHICAL ABSTRACT**

## Introduction

Diabetes mellitus is one of the most severe and incurable metabolic disorders, characterized by an increase in blood glucose level due to an absolute or relative lack of insulin and failure of insulin to act on its target tissue (Dey et al., 2015). Chronic hyperglycemia in diabetes is associated with long-term damage, dysfunction, and failure of various organs (Bajpay, 1993). Diabetes mellitus is known to cause hyperlipidemia through various metabolic derangements. Among several metabolic derangements, insulin deficiency has been known to stimulate lipolysis in the adipose tissue and give rise to hyperlipidemia (Ahmad et al., 2014). The number of people suffering from diabetes is increasing due to urbanization, demographic growth, aging, and growing incidences of obesity and lack of exercise (Badran and Laher, 2012). The existing drug therapies of sulfonylureas, biguanides, α-glucosidase inhibitors, and glinides have challenges in terms of cost, safety, and efficacy (Omonije et al., 2019). In particular, these hypoglycemic agents may cause adverse effects like renal toxicity, hypoglycemia, hepatotoxicity, and gastrointestinal disorders (Carpio and Fonseca, 2014).

Medicinal plants in this regard have become popular because of their perceived fewer side effects and many biological activities (Peiris et al., 2015). The antidiabetic medicinal plants may provide valuable sources for finding safer hypoglycemic agents Ethnobotanical statistics estimate that 1,200 rare plants worldwide may have antidiabetic potential (Arumugam et al., 2013)

*Leptopopus cordifolius* Decne (syn. *Andrachne cordifolia*) is a medicinal plant belonging to the family Phyllanthaceae, commonly found in the low hills of Northern hilly areas of Pakistan and the Himalayan region, including Azad Jammu and Kashmir. It is a small shrub, about 1 m tall, with ovate to elliptic leaves. It is distributed in Pakistan, Kashmir, India, and Nepal, on plains as well as on rocks, cliffs, wastelands, and on the bank of streams. The powder of the leaves of *L. cordifolius* is used for healing wounds and as a hypoglycemic agent (Ajaib, 2012; Munir and Qureshi, 2018). However, to the best of our knowledge, no scientific study documenting the antihyperglycemic and antihyperlipidemic behavior of *L. cordifolius* is available. Therfore, the current study was designed to investigate the antidiabetic ability of *L. cordifolius* in an alloxan-induced model of diabetic mice.

## Materials and Methods

### Collection and Processing of Plant

The *L. cordifolius* taxonomically identified leaves were collected from District Buner, northwest of Pakistan, after confirmation of its identification and authentication. The sample specimen with voucher number (AWKB-002) was deposited in the Herbarium of Botany Department Abdul Wali Khan University Mardan for future reference. The leaves of the plant were washed and air-dried for two weeks under shade. Using an electric grinder, they were ground to a uniform coarse powder and held in an airtight container at room temperature until it was used for extraction.

### Preparation of Extract

The powdered leaves of *L. cordifolius* were extracted by direct mollescence method using 70% methanol for 72°h. The extracts were weighed and the percentage yield was calculated by using the formula (David et al., 2017).

% Yield = Weight of the Crude Extract/Weight of Dried powdered sample ×100.

### Experimental Animals

The Swiss albino mice of 25––3°g weight and 3–4°weeks of age of either sex were utilized in the experiment. In compliance with OECD guidelines (Guideline, 2001), experimental animals were acclimatized to laboratory conditions for one week with free access during the experimental time to commercial pellet laboratory diets and water *ad* libitum (Council, 2010). The experimental animal’s procedures were used with the approval of an ethical committee of the Department of Pharmacy, Abdul Wali Khan University Mardan (0015/2019). Animals under ether anesthesia were killed and sacrificed at the end of the experiment.

### Diabetes Induction in Experimental Animals

Diabetes was provoked in overnight fasted mice by intraperitoneal injection of a single dose of alloxan monohydrate (150 mg/kg) (Szkudelski, 2001). The induction of diabetes was confirmed in mice 3 days after injection of alloxan by measuring the level of blood glucose. The animals with a blood glucose level greater than 200 mg/ml were considered as diabetic (Tzeng et al., 2014) and were designated for the study. The treatment with methanolic extracts, glibenclamide, and solvent fractions was initiated 72 h after alloxan injection to the mice.

### Acute Toxicity Study

An acute toxicity test was conducted in conjunction with the limit test recommended by Guideline 425 of the Organization for Economic Cooperation and Development (Guideline, 2001). The tests for acute toxicity were carried out using a fixed-dose protocol. Gross behavioral changes such as tremors, traction, salivation, loss of appetite, lacrimation, hair erection, diarrhea, mortality, and other symptoms of toxicity for 15°days were observed in the mice (Alema et al., 2020).

### Experimental Design

The mice were divided into nine groups consisting of six mice in each group. Group 1 served as normal control (saline, non-diabetic), group 2 served as alloxan (150°mg/kg)-treated control (diabetic control), group 3 received glibenclamide (10°mg/kg), group 4 and 5 received *L. cordifolius* methanolic extracts at the dose of 150 and 250°mg/kg respectively, whereas group 6, 7, 8, and 9 received n-hexane, chloroform, ethyl acetate, and aqueous fractions at the dose of 250 mg/kg orally. The treatment procedures lasted for 15 successive days. The extracts and saline solution were administered via oral gavage.

### Determination of Anti-diabetic Activity

The anti-diabetic ability of extracts and solvent fractions were assessed by an *in vivo* oral glucose tolerance test (OGTT) using albino mice at a dose of 150 and 250°mg/kg body weight. The dose of 150 and 250°mg/kg body weight were selected based on the effectiveness of their traditional claim (Shukla et al., 2011).

### Blood Glucose Determination

The blood glucose level of each mouse was determined by fractional tail amputation technique, taking blood from the tail vein by a one-touch electronic glucometer using glucose test strips (Akpan et al., 2012). The tails were then rubbed with ethanol to prevent infection.

### Body Weight Analysis

The body weight of the experimental animals was recorded before commencing the treatment (day 0) and throughout the experiment (day 7 and 15), and fluctuations in weight were recorded.

### Collection and Preparation of Blood Sample

Blood samples were collected from 24°h-fasted mice by cardiac puncture and retro-orbital plexus puncture method and were kept in sterile vials for 30°min for clotting. Serum samples were collected from clotted blood using a centrifuge operated at 3,000°rpm for 10 min (Chou et al., 2008; Parasuraman et al., 2015).

### Collection of Organs

The mice were anesthetized under mild ether and dissected. The tissue sample was taken for assessment of biochemical parameters (Li et al., 2008).

### Biochemical Analysis

The serum samples were then used for estimation of biochemical parameters such as alkaline phosphatase (ALP) (Sasaki, 1966), creatinine (Bowers, 1980), total cholesterol (TC) and triglycerides (TG) (Roeschlau et al., 1974), LDL (Friedewald et al., 1972), and HDL (Iwata et al., 1990).

The Serum Glutamate Pyruvate transaminase (SGPT) and Serum Glutamic Oxaloacetic transaminase (SGOT) activities were determined according to the method of Reitman and Frankel (Reitman and Frankel, 1957). These biochemical assays were measured by using commercial diagnostic kits.

### Histopathological Analysis

The liver, kidney, and pancreas of animals were expunged at the termination of the experiment and engrossed in 10% buffered formal-saline for histopathological investigations using Hematoxylin and Eosin (H&E) stains. The morphological changes in hepatic and pancreatic cells were examined under a microscope on albumenized glass slides, using 20× and 40× magnifications (Bancroft and Gamble, 2008).

### Antioxidant Enzyme Assay

The spectrophotometric measurement of antioxidant enzymatic activity of Superoxide dismutase SOD (Shah et al., 2013), peroxidase POD (Khan et al., 2015), and Catalase CAT (Khan et al., 2012) was carried out at 560, 420, and 230°nm respectively.

### Membrane Stabilization Assay

The membrane-stabilizing activity of the extracts was determined on human erythrocytes by collecting fresh blood samples from 10 volunteers and the absorbance was measured at 560°nm (Shinde et al., 1999; Omale and Okafor, 2008).

### GC-MS Profiling

A 20 mg sample of leaf powder was dissolved in 1°ml of methanol, vortexed for 5°min, sonicated for 10°min, and then centrifuged for 5°min at 12,000x°g. The supernatant was transferred into a fresh vial and was subjected (without any dilution) to GC-MS analysis.

GC-MS analysis of the active methanolic extract of *L. cordifolius* was carried out by using the GC-MS instrument (Model GCMS-QP2010 Ultra, Shimadzu Co., Japan) equipped with a capillary column DB-1 (0.25°*μ*m film × 0.25°mm i. d. ×30° m length). The instrument was operated in electron impact mode at ionization voltage (70°eV), injector temperature (230°C), and detector temperature (280°C). The carrier gas used was helium (99.9% purity) at a flow rate of 1 ml/min and about 1 *μ*L of the sample was injected. The tentative identification of compounds from the spectral data was based on the available mass spectral records (NIST and WILEY libraries) (Swamy et al., 2015).

### Statistical Analysis

All the values were expressed as mean ± Standard Error of the Mean (SEM). The results were analyzed for statistical significance using one-way ANOVA followed by Dunnett’s test with the aid of IBM Statistical Package for Social Scientist (SPSS-20). The level of significance was set at *p* < 0.05.

## Results

### Extraction Yield

The extraction yield of methanolic extract and different partitions of *L. cordifolius* showed that the extraction with methanol had the highest yield compared to the solvent extraction (Table 1).

**TABLE 1:** Extraction yield of *L. cordifolius*.

S.No	Plant extracts	Weight (g)	Percent yield (%)
1	Methanolic extract	430	91.48
2	n-Hexane	55	11.70
3	Chloroform	190	40.42
4	Ethyl acetate	58	12.34
5	Aqua	127	27.02

### Acute Toxicity Study

The extract was found benign up to a dose of 2000 mg/kg body weight from the safety data obtained. The behavior of the animals during the study was closely monitored and no lethality, behavioral changes, or mortality was noticed, which is regarded as a therapeutic benefit.

### Blood Glucose Level

The administration of the methanolic extract of *L. cordifolius* at 150 and 250°mg/kg significantly reduced (*p* < 0.01) the blood glucose level in diabetic mice at the end of the experiment in a dose dependent fashion, but less than glibenclamide-treated mice. Other extracts were not effective. (Table 2).

**TABLE 2:** Effects of *L. cordifolius* extracts on blood glucose level of alloxan-induced diabetic mice.

S.No	Treatments	Dose (mg/kg)	Blood glucose level (mg/dl)				
			Day 0	Day 4	Day 7	Day 10	Day 15
1	Normal Saline	0.3 ml	100.4 ± 2.7	98.4 ± 3.2	97.4 ± 1.9	96.4 ± 4.2	94 ± 3.6
2	Diabetic Control	0.3 ml	424.6 ± 2.7	438.8 ± 3.3	466.6 ± 2.7	474.8 ± 2.3	490 ± 2.7
3	Glibenclamide	10	431.4 ± 3.3	402.8* ± 2.8	360.6* ± 3.04	272.2** ± 4.9	206.6** ± 5.4
4	Methanolic Extract (1)	150	435.8 ± 4.4	412.6* ± 2.7	387.4* ± 3.6	346.6* ± 4.9	304.2* ± 2.8
5	Methanolic Extract (2)	250	431.2 ± 3.9	408* ± 3.16	360.8* ± 3.1	320.8* ± 2.8	270.4** ± 4.5
6	n-Hexane	250	459.2 ± 3.4	458.2 ± 4.2	449.4 ± 3.8	454.6 ± 4.5	466.8 ± 2.16
7	Chloroform	250	455.6 ± 3.6	453.8 ± 3.4	440.4 ± 3.04	429.6 ± 3.36	414.2 ± 4.3
8	Ethyl acetate	250	419.8 ± 3.03	417.4 ± 3.04	421.2 ± 4.08	425.6 ± 2.4	440 ± 4.4
9	Aqua	250	414 ± 2.9	412.6 ± 3.6	412 ± 2.8	433.4 ± 4.03	456.2 ± 1.9

Values are expressed as mean ± SEM n = 6 in each group. Data analyzed by ONE WAY ANOVA followed by 187 Dunnett’s multiple comparisons test. * P < 0.05; **P < 0.01; compared with diabetic control.

### Changes in Body Weight and Fasting Blood Glucose Level

The methanolic extracts at the dose of 150 and 250 significantly improved the body weight (*p* < 0.05; *p* < 0.01) in alloxanised diabetic mice and produced maximum fall (*p* < 0.01) in the FBG levels of diabetic mice after 15 days of treatment (Table 3). Other extracts were not effective.

**TABLE 3:** Effects of *L. cordifolius* extracts on body weight of mice.

S.No	Treatments	Dose (mg/kg)	Body weight (g)				
			Day 0	Day 4	Day 7	Day 10	Days 15
1	Normal Saline	0.3 ml	22.2 ± 0.28	23.6 ± 0.20	24.6 ± 0.25	26.1 ± 0.47	26.8 ± 0.45
2	Diabetic Control	0.3 ml	29.2 ± 0.49	28.6 ± 0.38	26.3 ± 0.49	25.4 ± 0.38	24.5 ± 0.33
3	Glibenclamide	10	30.3 ± 0.28	31.9 ± 0.38	32.8* ± 0.43	33.5* ± 0.31	34.9** ± 0.22
4	Methanolic Extract(1)	150	30.8 ± 0.27	31.7 ± 0.54	32.4* ± 0.36	32.6 ± 0.53	33.1* ± 0.36
5	Methanolic Extract(2)	250	29.3 ± 0.33	30.6 ± 0.93	32.4* ± 0.36	33.1* ± 0.41	34.6** ± 0.27
6	n-Hexane	250	28.8 ± 0.27	28.2 ± 0.28	27.5 ± 0.24	27 ± 0.27	26.2 ± 0.31
7	Chloroform	250	27.5 ± 0.28	27.3 ± 0.36	28.2 ± 0.16	28.4 ± 0.22	29.2 ± 0.20
8	Ethyl acetate	250	29.5 ± 0.29	28.5 ± 0.31	27.6 ± 0.23	26.6 ± 0.22	25.7 ± 0.27
9	Aqua	250	29.2 ± 0.20	28.6 ± 0.19	27.8 ± 0.20	26.7 ± 0.20	25.5 ± 0.27

Values are expressed as mean ± SEM n = 6 in each group. Data analyzed by ONE WAY ANOVA followed by Dunnett’s multiple comparisons test. * P < 0.05; **P < 0.01; compared with diabetic control.

### Lipid Profile

The lipid profile in diabetic mice was severely disturbed in diabetic mice by alloxan administration when compared with the control group. The methanolic extract at the dose of 150 and 250°mg/kg of *L. cordifolius* significantly reduced the levels of lipid parameters to near normal (Table 4). Other extracts were not effective.

**TABLE 4:** Effects of *L. cordifolius* extracts on lipid profile parameters.

S.No	Treatments	Dose (mg/kg)	TC (mg/dl)	TG (mg/dl)	LDL (mg/dl)	HDL (mg/dl)
1	Normal Saline	0.3 ml	181.3 ± 1.45	163.3 ± 4.7	173 ± 1.1	35 ± 0.5
2	Diabetic Control	0.3 ml	183.4 ± 1.45	167.6 ± 1.76	161.9 ± 12.1	36.6 ± 0.4
3	Glibenclamide	10	136.4** ± 0.9	145.8** ± 0.7	125.4** ± 0.2	43.4** ± 0.5
4	Methanolic Extract(1)	150	165.1 ± 3.52	154.8 ± 1.9	142.3 ± 0.3	40.9 ± 1.6
5	Methanolic Extract(2)	250	154.7** ± 3.76	148** ± 1.7	128.6** ± 0.7	41.2** ± 1.2
6	n-Hexane	250	178.4 ± 1.90	164.3 ± 0.6	147.4 ± 0.4	35.6 ± 0.7
7	Chloroform	250	171.5 ± 5.7	165.5 ± 3.7	143.7 ± 0.4	37.3 ± 0.6
8	Ethyl acetate	250	178.4 ± 1.81	165.3 ± 0.7	147.3 ± 0.6	35.8 ± 0.9
9	Aqua	250	178.8 ± 1.11	166.3 ± 1.03	148.4 ± 0.7	35.2 ± 0.4

Values are expressed as mean ± SEM n = 6 in each group. Data analyzed by ONE WAY ANOVA followed by Dunnett’s multiple comparisons test. * P < 0.05; **P < 0.01; compared with diabetic control.

### Serum Profile

A significant elevation in SGPT, SGOT, ALP, and creatinine was observed in alloxan-induced diabetic mice when compared to normal mice. Oral administration of *L. cordifolius* leaves, methanolic extract 150 and 250°mg/kg, and glibenclamide treatment significantly improved the above parameters (Table 5).

**TABLE 5:** Effects of extracts of *L. cordifolius* on serum biochemical parameters.

S.No	Treatments	Dose (mg/kg)	SGPT (U/I)	SGOT (U/I)	ALP (U/I)	Creatinine (mg/dl)
1	Normal Saline	0.03 ml	19.3 ± 2.08	23.4 ± 0.65	173.6 ± 6.77	1.05 ± 0.31
2	Diabetic Control	0.03 ml	48.4 ± 1.45	43.6 ± 1.45	331.3 ± 1.76	2.3 ± 0.08
3	Glibenclamide	0.03 ml	32.4** ± 0.36	25.2** ± 0.60	169.8** ± 1.44	0.98** ± 0.05
4	Methanolic Extract (1)	150	29.3 ± 2.86	30.5 ± 0.57	280 ± 1.41	1.87 ± 0.03
5	Methanolic Extract (2)	250	27.8** ± 2.60	26.4** ± 2.15	231.1** ± 2.25	1.26** ± 0.22
6	n-Hexane	250	37.3 ± 1.44	32.2 ± 0.17	280.9 ± 11.5	1.88 ± 0.02
7	Chloroform	250	33.5 ± 0.92	29.4 ± 1.15	270 ± 29.6	1.84 ± 0.04
8	Ethyl acetate	250	38.2 ± 1.43	34.2 ± 0.78	286.6 ± 15.5	1.90 ± 0.02
9	Aqua	250	35.9 ± 1.18	33.1 ± 0.31	296.1 ± 8.93	1.93 ± 0.02

Values are expressed as mean ± SEM; n = 6 in each group. ^∗∗^P < 0.01 as compared with diabetic control at the same time (one-way ANOVA followed by Dunnett’s multiple comparison test). TC, total cholesterol; TG, triglycerides; LDL, low density lipids and HDL, high density lipids.

### Antioxidant Enzymes

Diabetes mellitus significantly reduced antioxidant enzymes, like catalases (CAT), peroxidases (POD), and superoxide dismutase (SOD) levels, and elevated the action of reactive oxygen species. The methanolic extract at the dose of 150 and 250°mg/kg significantly increased the activities of antioxidant enzymes. However, other extracts treated group showed no significant difference in comparison to the glibenclamide-treated group (Table 6).

**TABLE 6:** Determination of antioxidant enzymes activity.

S. No	Treatments	Dose (mg/kg)	SOD (U/mg)	POD (U/mg)	Catalase (U/mg)
1	Normal Saline	0.03 ml	4.87 ± 0.24	6.27 ± 0.23	9.38 ± 0.11
2	Diabetic Control	0.03 ml	3.72 ± 0.04	5.97 ± 0.02	8.63 ± 0.03
3	Glibenclamide	0.03 ml	2.02** ± 0.03	4.80** ± 0.03	4.57** ± 0.40
4	Methanolic Extract (1)	150	2.98* ± 0.03	3.90* ± 0.22	5.04* ± 0.26
5	Methanolic Extract (2)	250	2.66 ** ± 0.10	4.62** ± 0.09	4.94** ± 0.60
6	n-Hexane	250	3.41 ± 0.12	2.86 ± 0.03	5.62 ± 0.32
7	Chloroform	250	3.71 ± 0.21	3.32* ± 0.27	5.41 ± 0.06
8	Ethyl acetate	250	3.12 ± 0.18	2.66 ± 0.14	5.91 ± 0.26
9	Aqua	250	2.95 ± 0.23	2.36 ± 0.17	5.85 ± 0.03

Values are expressed as mean ± SEM; n = 6 in each group. ^∗∗^P < 0.01 as compared with diabetic control at the same time (one-way ANOVA followed by Dunnett’s multiple comparison test). SGPT, Serum Glutamate Pyruvate Transaminase; SGOT, Serum Glutamic Oxaloacetic Transaminase; ALP, alkaline phosphatase.

### Membrane Stabilizing Activity

In the hypotonic-solution-induced condition, methanolic extract showed the highest protection of erythrocyte membrane (76%), which was comparable to haemolysis inhibited by standard acetyl salicylic acid (Table 7).

**TABLE 7:** Thrombolytic activity of *A. cordifolia* extract/fractions.

S.No	Extract/Fractions	Concentration (mg/ml)	Thrombolytic activity	% Inhibition
1	Methanolic	1	0.1678 ± 0.06	76
2	n-Hexane	1	0.6016 ± 0.03	14.6
3	Chloroform	1	0.4411 ± 0.05	37.4
4	Ethyl acetate	1	0.5016 ± 0.04	28.8
5	Aqueous	1	0.5737 ± 0.19	18.6
6	Acetyl salicylic acid (Control)	0.10	0.705 ± 0.06	86

Values are expressed as mean ± SEM. Data analyzed by ONE WAY ANOVA followed by Dunnett’s multiple comparisons test. * P < 0.05; **P < 0.01, compared with diabetic control. Data are expressed as mean ± SD (n = 3).

### GC-MS Analysis

**FIGURE 1:**
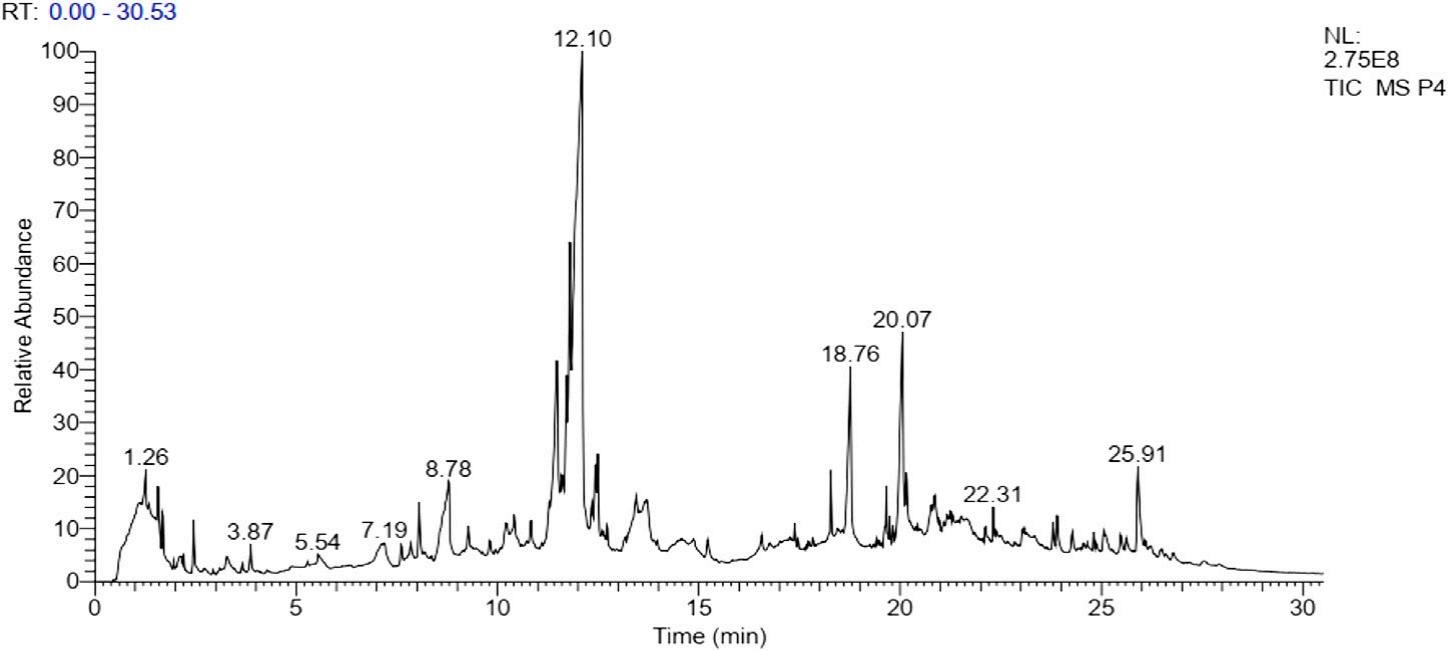
GC-MS chromatogram of *L. cordifolius* extract.

The results of GC-MS analysis revealed the tentative identity of 52 compounds present in methanolic extract *L. cordifolius*. The active principles with their retention time, probability, molecular weight, and concentration (peak area %) are presented in Table 8 and Figure 1.

**TABLE 8:** Chemical constituents from GC-MS analysis of methanolic extract of *L. cordifolius*.

S.No	Compound Name	Area %	RT	Probability	Molecular formula	MW
1	Amyl Nitrite	13.62	1.24	12.42	C_5_H_11_NO_2_	117
2	Pentane, 3-methyl	13.62	1.24	11.46	C_5_H_11_NO_2_	86
3	Butyl glycolate	13.62	1.24	11.46	C_6_H_12_O_3_	132
4	Furfural	0.38	2.14	52.91	C_5_H_4_O_2_	96
5	3-Furaldehyde	0.38	2.14	37.35	C_5_H_4_O_2_	96
6	2-Furanmethanol	0.55	2.47	54.29	C_5_H_6_O_2_	98
7	[1,3,4]Thiadiazol,2-amino-5-(2-piperidin-1-ylethyl)-	0.69	3.30	8.96	C_9_H_16_N_4_S	212
8	1-Piperidineethanol	0.69	3.30	6.14	C_7_H_15_NO	129
9	4H-Pyran-4-one, 2, 3-dihydro-3,5-dihydroxy-6-methyl-	0.52	3.87	17.90	C_6_H_8_O_4_	144
10	2-Propyl-tetrahydropyran-3-ol	0.52	3.87	10.42	C_8_H_16_O_2_	144
11	Deoxyspergualin	0.03	4.30	21.53	C_17_H_37_N_7_O_3_	387
12	1,3,9-Trioxaspiro[5.5]undecane	0.33	4.93	18.62	C_8_H_14_O_3_	158
13	2,5-Dimethyl-4-hydroxy-3(2H)-furanone	0.92	5.58	31.53	C_6_H_8_O_3_	128
14	3-Propylnorleucine	4.32	5.58	4.32	C_9_H_19_NO_2_	173
15	a-D-Glucopyranoside	0.08	6.30	33.47	C_18_H_32_O_16_	504
16	Desulphosinigrin	0.08	6.30	9.84	C_10_H_17_NO_6_S	279
17	4H-Pyran-4-one, 2,3-dihydro-3,5-dihydroxy-6-methyl	1.59	7.13	92.67	C_6_H_8_O_4_	144
18	2-Hydroxy-4,6-dimethylbenzaldehyde	1.75	8.07	9.59	C_9_H_10_O_2_	150
19	2-Furancarboxaldehyde, 5-(hydroxymethyl)-	3.49	8.74	94.95	C_6_H_6_O_3_	126
20	2-Methoxy-4-vinylphenol	0.92	9.27	11.48	C_9_H_10_O_2_	150
21	α-D-Glucopyranosyl-(1->3)-β-D-fructofuranosyl β-D-glucopyranoside	0.11	9.82	30.85	C_18_H_32_O_16_	504
22	d-Mannose	0.11	9.82	14.05	C_6_H_12_O_6_	180
23	Ethaneperoxoic acid	1.89	10.39	10.90	C_12_H_13_NO_3_	219
24	Furan, 2-(2-furanylmethyl)-5-methyl-	0.57	10.82	29.36	C_10_H_10_O_2_	162
25	Decanal	5.80	11.45	5.31	C_10_H_20_O	156
26	1,12-Dodecanediol	5.80	11.45	4.28	C_12_H_26_O_2_	202
27	1,10-Decanediol	29.35	12.02	13.90	C_10_H_22_O_2_	174
28	Z-10-Pentadecen-1-ol	1.74	12.45	8.65	C_15_H_30_O	226
29	2H-Pyran-2-one, tetrahydro-4-hydroxy-6-pentyl	5.53	13.67	14.68	C_10_H_18_O_3_	186
30	9-Hexadecenoic acid	1.16	14.59	39.48	C_16_H_30_O_2_	254
31	Tetraacetyl-D-xylonic nitrile	0.83	14.83	7.66	C_14_H_17_NO_9_	343
32	Oleic Acid	0.39	15.22	11.04	C_18_H_34_O_2_	282
33	Tetradecanoic acid	1.04	16.54	73.29	C_14_H_28_O_2_	228
34	l-Gala-l-ido-octonic lactone	1.45	17.34	20.87	C_8_H_14_O_8_	238
35	Cholestan-3-ol, 2-methylene-	0.09	17.78	44.09	C_28_H_48_O	400
36	Hexadecanoic acid, methyl ester	1.02	18.29	59.66	C_17_H_34_O_2_	270
37	n-Hexadecanoic acid	4.57	18.74	75.24	C_16_H_32_O_2_	256
38	7-Methyl-Z-tetradecen-1-ol acetate	0.02	19.41	56.16	C_17_H_32_O_2_	268
39	9,12-Octadecadienoyl chloride	0.59	19.66	15.80	C_18_H_31_ClO	298
40	l-Gala-l-ido-octonic lactone	1.45	17.34	20.87	C_18_H_32_O_16_	504
41	10-Methyl-8-tetradecen-1-ol acetate	1.59	20.84	26.48	C_17_H_32_O_2_	268
42	Dasycarpidan-1-methanol, acetate	1.20	21.25	35.93	C_20_H_26_N_2_O_2_	326
43	9,12,15-Octadecatrienoic acid, 2,3-dihydroxypropyl ester	1.29	23.10	47.72	C_21_H_36_O_4_	352
44	8-(2-Aminoethylthio)guanosine-3′,5′-cyclic monophosphate	0.83	23.88	97.72	C_12_H_16_N_6_O_7_PS	419
45	Palmitic acid, 9-hexadecenyl ester, (Z)-	0.32	24.28	11.27	C_32_H_62_O_2_	478
46	Vitamin E	1.20	25.08	55.93	C_29_H_50_O_2_	430
47	β-sitosterol	3.11	25.93	42.59	C_29_H_50_O	414
48	Stigmasterol, 22,23-dihydro-	3.11	25.93	19.97	C_29_H_50_O	414
49	Olean-13(18)-ene	0.55	26.50	41.48	C_30_H_50_	410
50	Carotene, 1,1′,2,2′-tetrahydro-1,1′-dimethoxy	0.01	27.19	61.58	C_42_H_64_O2	600
51	Ergost-8(14)-en-3-ol	0.01	27.19	11.54	C_28_H_48_O	400
52	Betulin	0.15	27.91	11.94	C_30_H_50_O_2_	442

RT, Retention time; MW, Molecular weight.

## Discussion

The current research study investigated the antidiabetic and antihyperlipidemic effects of the methanolic extracts and solvent fractions of *L. cordifolius* in alloxan-induced diabetic mice. Alloxan is a cytotoxic glycoside and acts as a diabetogenic by the annihilation of the islets of Langerhans and causes an enormous decline in insulin discharge, thus prompting hyperglycemia (Grover et al., 2000). Glibenclamide is used as a standard antidiabetic drug in alloxan-induced mild diabetes to compare the antidiabetic effects of a variety of bioactive compounds (Ramkumar et al., 2011). The extracts were found to be safe up to the dose of 2000 mg/kg in normal healthy mice from the safety data obtained (Yehya et al., 2019).

Alloxan induces diabetes through ROS that leads to rapid destruction of pancreatic beta cells, causing hyperglycemia (Stanely et al., 2000). Hyperglycemia in turn increases the generation of free radicals by glucose auto-oxidation (Bajaj and Khan, 2012). In the current research study, oral administration of *L. cordifolius* methanolic extract of 150 and 250 mg/kg body weight to diabetic mice for 15°days significantly reduced the fasting blood glucose and glycerides were reduced only at 250°mg/k, compared with diabetic control mice. In support of our study, the *L. cordifolius* methanolic extract (250°mg/kg body weight) cause their anti-hyperglycemic activity by augmenting the production of insulin or regenerating pancreatic cells (Patel et al., 2012). Although the effective doses of 150 and 250°mg/kg reported here are comparable with other antidiabetic plants (Ghazanfar et al., 2014; Alema et al., 2020), they are at the upper end of the dose range and further research needs to be done to concentrate the active principles and thereby reduce the effective dose. Unfortunately, the results with different solvents did not achieve this result as they were less effective than methanol. The observed decline in body weight of diabetic mice could be attributed to the surging body mass index and the progression of skeletal size and epididymis fats (Wu and Huan, 2008). However, treatments with orally administered methanolic, and chloroform extracts of 250°mg/kg significantly improved the body weight compared with diabetic control, which signifies its protective effect in controlling muscle wasting i.e. reversal of gluconeogenesis (Mestry et al., 2017). In diabetes mellitus, hyperlipidemia occurs as a result of the excess mobilization of fats from the adipose tissue due to the underutilization of glucose (Akpan et al., 2012). Hyperglycemia is accompanied by a rise in TC, TG, and LDL, and a fall in HDL levels (Gao et al., 2009). The methanolic extract *L. cordifolius* and gibenclamide might act on reserved fats and inhibit the release of free fatty acids, decreasing the total cholesterol and triglyceride levels, and increasing the HDL level.

It is acknowledged that diabetes is linked to irreversible hepatic damage leading to the proliferation of different enzymes including SGOT, SGPT, ALP, and creatinine in the blood (Pari and Latha, 2002; Preethi and Kuttan, 2009). *L. cordifolius* methanolic extract significantly reduced the serum creatinine, thus treating and preventing the progression of renal damage in diabetic mice. Reports have shown that the reversal of such changes in the above parameters is allied with the presence of bioactive constituents in the extract (Lal et al., 2009; Sharma et al., 2019). In diabetes mellitus, high glucose can inactivate antioxidant enzymes SOD, POD, and, CAT by glycating these proteins, thus producing induced oxidative stress, which in turn causes lipid peroxidation. The *L. cordifolius* methanolic extract increased the activities of SOD, POD, and CAT, thus maintaining the antioxidant status in the erythrocytes of diabetic mice. Several studies revealed that injury to membranes of RBCs due to hyperglycemia makes cells more susceptible to secondary damage through lipid peroxidation, haemolysis, and oxidation of haemoglobin (Halliwell, 1999). The inhibition of the erythrocyte lysis property of *L. cordifolius* could be the possible mechanism for its anti-diabetic activity. It may also be due to the presence of plant biochemical compounds in the extract which exert a profound stabilizing effect on lysosomal membranes and their cation-binding ability (Oyedapo et al., 2004).

Various secondary metabolites and phytochemicals isolated from diverse plant species have been found to have potent anti-hyperglycemic and glucose suppressive effects (Sharma et al., 2010). The secondary metabolites which were tentatively identified in *L. cordifolius* crude methanolic extract may be accountable for its observed glucose suppressive and anti-hyperglycemic activity, due to its ability to stimulate glycogenesis in the liver, release insulin from pancreatic ß-cells, or inhibit glucose absorption in the gut (Sezik et al., 2005).

Several studies revealed that injury to membranes of RBCs makes cells more susceptible to secondary damage through lipid peroxidation, haemolysis, and oxidation of haemoglobin (Halliwell and Whiteman, 2004). The inhibition of the erythrocyte lysis property of *L. cordifolius* could also be the possible mechanism for its anti-diabetic activity (Chaitanya et al., 2011). GC-MS analysis of methanolic extract of *L. cordifolius* tentatively identified the presence of various bioactive compounds with different retention times. The tentatively identified compounds are known to possess several biological and pharmacological activities (Grover et al., 2002; Kumar et al., 2010; Hema et al., 2011). Several studies have shown that ß-Sitosterol, stigmasterol, betulin, ergost-8(14)-en-3-ol, n-hexadecanoic acid, and palmitic acid have been found to have hypoglycemic effects by reducing the absorptions of cholesterol from the gut as shown in the Table 9 (Ikeda et al., 1988; Rajasekaran et al., 2004).

**TABLE 9:** Antidiabetic compounds identified from GC-MS.

S.NO	Compounds	Compounds nature	References
1	Betulin	Triterpene	Birgani et al. (2018)
2	Ergost-8(14)-en-3-ol	Steroid	Luo et al. (2010)
3	Vitamin E	Vitamin	Bharti et al. (2012)
4	Stigmasterol	Steroid	Panda et al. (2009), Gabay et al. (2010), Tyagi and Agarwal (2017)
5	a-D-Glucopyranoside, O-a-D-glucopyranosyl-(1.fwdarw.3)-a-D –fructofuranosyl	Steroid	Kadhim et al. (2017), Al-Gara et al. (2019)
6	l-Gala-l-ido-octonic lactone	Steroid	Kadhim et al. (2017)
7	Dasycarpidan-1-methanol, acetate	Ester	Al-Gara et al. (2019)
8	β-sitosterol	Steroid	Gupta et al. (2011)
9	d-Mannose	Sugar	van de Venter et al. (2008)
10	n-hexadecenoic acid	Ester	Parker et al. (2003)
11	13-Octadecenoic acid	Ester	Su et al. (2013)
12	2-Methoxy-4-vinylphenol	Phenolic	Vadivel and Gopalakrishnan (2011)

## Conclusion

The current research study demonstrated the first pharmacological insight into the antioxidant, antidiabetic, and antihyperlipidemic potential of the *L. cordifolius.* Based on the results exhibited by this study, it is concluded that *L. cordifolius* methanolic extract had significant anti-hyperglycemic activity in alloxan-induced diabetic mice compared with that of glibenclamide, as evident from the restoration of blood glucose levels, thus validating its traditional utilization. Future research needs to focus on bioassay guided isolation and confirmed identification of the active principles using standard or spectral workup. This could inform better extraction and formulation procedures to lower the dose required, as well as lead to a validated phytochemical analysis of a standardized product. Further study of the mode of action of active principles is warranted.

## Data Availability

The original contributions presented in the study are included in the article/Supplementary Material, further inquiries can be directed to the corresponding author.

## References

[B1] Ahmad W. Khan I. Khan M. A. Ahmad M. Subhan F. Karim N. (2014). Evaluation of antidiabetic and antihyperlipidemic activity of Artemisia indica linn (aeriel parts) in Streptozotocin induced diabetic rats. J. Ethnopharmacol. 151, 618–623. 10.1016/j.jep.2013.11.012 24252495

[B2] Ajaib M. (2012). Exploration of floral diversity of District kotli (Azad Jammu & Kashmir) and evaluation of ethnopharmacological effects of some medicinal plants of the area. Pakistan: GC University Lahore.

[B3] Akpan E. J. Okokon J. E. Offong E. (2012). Antidiabetic and hypolipidemic activities of ethanolic leaf extract and fractions of Melanthera scandens. Asian Pac. J. Trop. Biomed. 2, 523–527. 10.1016/S2221-1691(12)60089-6 23569963 PMC3609341

[B4] Al-Gara N. I. Abu-Serag N. A. Shaheed K. A. A. Al Bahadly Z. K. (2019). “Analysis of bioactive phytochemical compound of (Cyperus alternifolius L.) by using gas chromatography–mass spectrometry,” in IOP conference series: Mater. Sci. Eng. April 15, 2019, Iraq (United Kingdom: IOP Publishing), Vol. 571, 012047.

[B5] Alema N. M. Periasamy G. Sibhat G. G. Tekulu G. H. Hiben M. G. (2020). Antidiabetic activity of extracts of Terminalia brownii fresen. Stem bark in mice. J. Exp. Pharmacol. 12, 61. 10.2147/JEP.S240266 32110120 PMC7039073

[B6] Arumugam G. Manjula P. Paari N. (2013). A review: anti diabetic medicinal plants used for diabetes mellitus. J. Acute Dis. 2, 196–200. 10.1016/s2221-6189(13)60126-2

[B7] Badran M. Laher I. (2012). Type II diabetes mellitus in Arabic-speaking countries. Int. J. Endocrinol. 2012, 902873. 10.1155/2012/902873 22851968 PMC3407623

[B8] Bajaj S. Khan A. (2012). Antioxidants and diabetes. Indian J. Endocrinol. Metab. 16, S267–S271. 10.4103/2230-8210.104057 23565396 PMC3603044

[B9] Bajpay A. (1993). Ecological Studies of Boerhaavia verticillata poir with special reference to phytochemical and therapeutic importance. PhD thesis. Varanasi, India: Banaras Hindu University.

[B10] Bancroft J. D. Gamble M. (2008). “Theory and practice of histological techniques,” in Journal of Neuropathology and Experimental Neurology. Editors Livingstone C. Philadelphia E. (California: Elsevier health sciences), Vol. 67, 633.

[B11] Bharti S. K. Krishnan S. Kumar A. Rajak K. K. Murari K. (2012). Antihyperglycemic activity with DPP-IV inhibition of alkaloids from seed extract of *Castanospermum australe*: Investigation by experimental validation and molecular docking. Phytomed. 20, 24–31. 10.1016/j.phymed.2012.09.009 23063145

[B12] Birgani G. A. Ahangarpour A. Khorsandi L. Moghaddam H. F. (2018). Anti-diabetic effect of betulinic acid on streptozotocin-nicotinamide induced diabetic male mouse model. Braz. J. Pharm. Sci. 54. 10.1590/s2175-97902018000217171

[B13] Bowers L. D. (1980). Kinetic serum creatinine assays I. The role of various factors in determining specificity. Clin. Chem. 26, 551–554. 10.1093/clinchem/26.5.0551 7261300

[B14] Carpio G. R. A. Fonseca V. A. (2014). Update on safety issues related to antihyperglycemic therapy. Diabetes Spectr. 27, 92–100. 10.2337/diaspect.27.2.92 26246765 PMC4522884

[B15] Chaitanya R. Sandhya S. David B. Vinod K. Murali S. (2011). HRBC membrane stabilizing property of roor, stem and leaf of glochidion velutinum. Int. J. Res. Pharmaceut. Biomed. Sci. 2, 256–259.

[B16] Chou Y. C. Tsai Y. C. Chen C. M. Chen S. M. Lee J. A. (2008). Determination of lipoprotein lipase activity in post heparin plasma of streptozotocin‐induced diabetic rats by high‐performance liquid chromatography with fluorescence detection. Biomed. Chromatogr. 22, 502–510. 10.1002/bmc.960 18205134

[B17] Council N. R. (2010). Guide for the care and use of laboratory animals. Washington, DC: National Academies Press.

[B18] David J. Afolabi E. Olotu P. Ojerinde S. Agwom F. Ajima U. (2017). Phytochemical analysis, antidiabetic and toxicity studies of the methanolic leaf extract of Detarium microcarpum guill and perr in wistar albino rats. Jaipur: Chandra Shekhar Sharma.

[B19] Dey P. Saha M. R. Chowdhuri S. R. Sen A. Sarkar M. P. Haldar B. (2015). Assessment of anti-diabetic activity of an ethnopharmacological plant Nerium oleander through alloxan induced diabetes in mice. J. Ethnopharmacol. 161, 128–137. 10.1016/j.jep.2014.12.012 25498854

[B20] Friedewald W. T Levy R. I. Fredrickson D. S. (1972). Estimation of the concentration of low density lipoprotein cholesterol in plasma, without the use of the preparative ultracentrifuge. Clin. Chem. 18, 499–502. 10.1093/clinchem/18.6.499 4337382

[B21] Gabay O. Sanchez C. Salvat C. Chevy F. Breton M. (2010). Stigmasterol: a phytosterol with potential anti-osteoarthritic properties. Osteoarthr. Cartil. 18, 106–116. 10.1016/j.joca.2009.08.019 19786147

[B22] Gao D. Li Q. Li Y. Liu Z. Fan Y. Liu Z. (2009). Antidiabetic and antioxidant effects of oleanolic acid from Ligustrum lucidum Ait in alloxan‐induced diabetic rats. Phytother. Res. 23, 1257–1262. 10.1002/ptr.2603 19274687

[B23] Ghazanfar K. Ganai B. A. Akbar S. Mubashir K. Dar S. A. Dar M. Y (2014). Antidiabetic activity of Artemisia amygdalina Decne in streptozotocin induced diabetic rats. Biomed. Res. Int. 2014, 185676. 10.1155/2014/185676 24967338 PMC4055220

[B24] Grover J. Vats V. Rathi S. (2000). Anti-hyperglycemic effect of Eugenia jambolana and Tinospora cordifolia in experimental diabetes and their effects on key metabolic enzymes involved in carbohydrate metabolism. J. Ethnopharmacol. 73, 461–470. 10.1016/s0378-8741(00)00319-6 11091000

[B25] Grover J. Yadav S. Vats V. (2002). Medicinal plants of India with anti-diabetic potential. J. Ethnopharmacol. 81, 81–100. 10.1016/s0378-8741(02)00059-4 12020931

[B26] Guideline O. (2001). “Acute oral toxicity up and-down procedure,” in OECD guidelines for the testing of chemicals (Paris, OECD: Organization for Economic Cooperation and Development), Vol. 425, 27.

[B27] Gupta R. Sharma A. K. Dobhal M. Sharma M. Gupta R. (2011). Antidiabetic and antioxidant potential of β‐sitosterol in streptozotocin‐induced experimental hyperglycemia. J. Diabetes 3, 29–37. 10.1111/j.1753-0407.2010.00107.x 21143769

[B28] Halliwell B. (1999). Antioxidant defenses. Free Radic. Biol. Med. 31, 4 261–272. 10.1080/10715769900300841 10517532

[B29] Halliwell B. Whiteman M. (2004). Measuring reactive species and oxidative damage *in vivo* and in cell culture: how should you do it and what do the results mean?. Br. J. Pharmacol. 142, 231–255. 10.1038/sj.bjp.0705776 15155533 PMC1574951

[B30] Hema R. Kumaravel S. Alagusundaram K. (2011). GC/MS determination of bioactive components of Murraya koenigii. J. Am. Sci. 7, 80–83.

[B31] Ikeda I. Tanaka K. Sugano M. Vahouny G. Gallo L. (1988). Inhibition of cholesterol absorption in rats by plant sterols. J. Lipid Res. 29, 1573–1582. 10.1016/s0022-2275(20)38403-0 2468730

[B32] Iwata K. Inayama T. Kato T. (1990). Effects of Spirulina platensis on plasma lipoprotein lipase activity in fructose-induced hyperlipidemic rats. J. Nutr. Sci. Vitaminol. 36, 165–171. 10.3177/jnsv.36.165 2117648

[B33] Kadhim M. J. Al-Rubaye A. F. Hameed I. H. (2017). Determination of bioactive compounds of methanolic extract of vitis vinifera using GC-MS. Int. J. Toxicol. Pharmacol. Res. 9, 113–126. 10.25258/ijtpr.v9i02.9047

[B35] Khan R. A. Khan M. R. Sahreen S. Shah N. A. (2012). Hepatoprotective activity of *Sonchus asper* against carbon tetrachloride-induced injuries in male rats: a randomized controlled trial. BMC Complement. Altern. Med. 12, 90. 10.1186/1472-6882-12-90 22776436 PMC3457902

[B36] Khan R. A. Khan M. R. Shah N. A. Sahreen S. Siddiq P. (2015). Modulation of carbon tetrachloride-induced nephrotoxicity in rats by n-hexane extract of *Sonchus asper* . Toxicol. Ind. Health 31, 955–959. 10.1177/0748233713485885 23589407

[B37] Kumar P. P. Kumaravel S. Lalitha C. (2010). Screening of antioxidant activity, total phenolics and GC-MS study of Vitex negundo. Afr. J. Biochem. Res. 4, 191–195. 10.5897/AJBR.9000213

[B38] Lal S. S. Sukla Y. Singh A. Andriyas E. A. Lall A. M. (2009). Hyperuricemia, high serum urea and hypoproteinemia are the risk factor for diabetes. Asia J. Med. Sci. 1, 33–34.

[B39] Li M. Kim D. H. Tsenovoy P. L. Peterson S. J. Rezzani R. Rodella L. F. (2008). Treatment of obese diabetic mice with a heme oxygenase inducer reduces visceral and subcutaneous adiposity, increases adiponectin levels, and improves insulin sensitivity and glucose tolerance. Diabetes 57, 1526–1535. 10.2337/db07-1764 18375438

[B40] Luo C. Zhang W. Sheng C. Zheng C. Yao J. Miao Z. (2010). Chemical composition and antidiabetic activity of Opuntia Milpa Alta extracts. Chem. Biodivers. 7, 2869–2879. 10.1002/cbdv.201000077 21161999

[B41] Mestry S. N. Dhodi J. B. Kumbhar S. B. Juvekar A. R. (2017). Attenuation of diabetic nephropathy in streptozotocin-induced diabetic rats by Punica granatum Linn. leaves extract. J. Traditional Complement. Med. 7, 273–280. 10.1016/j.jtcme.2016.06.008 PMC5506633 28725620

[B42] Munir M. Qureshi R. (2018). “Antidiabetic plants of Pakistan,” in Plant and human health Editors M. Ozturk and K. R. Hakeem (Springer), Vol. 1, 463–545.

[B43] Omale J. Okafor P. N. (2008). Comparative antioxidant capacity, membrane stabilization, polyphenol composition and cytotoxicity of the leaf and stem of Cissus multistriata. Afr. J. Biotechnol. 7 (17), 29–33. 10.4314/ajb.v7i17.59240

[B44] Omonije O. O. Saidu A. N. Muhammad H. L. (2019). Anti-diabetic activities of Chromolaena odorata methanol root extract and its attenuation effect on diabetic induced hepatorenal impairments in rats. Clin. Phytoscience 5, 23. 10.1186/s40816-019-0115-1

[B45] Oyedapo O. Akinpelu B. Orefuwa S. (2004). Anti-inflammatory effect of Theobroma cacao, root extract. J. Trop. Med. Plants 5, 161–166.

[B46] Panda S. Jafri M. Kar A. Meheta B. (2009). Thyroid inhibitory, antiperoxidative and hypoglycemic effects of stigmasterol isolated from Butea monosperma. Fitoterapia 80, 123–126. 10.1016/j.fitote.2008.12.002 19105977

[B47] Parasuraman S. Balamurugan S. Christapher P. V. Petchi R. R. Yeng W. Y. (2015). Evaluation of antidiabetic and antihyperlipidemic effects of hydroalcoholic extract of leaves of Ocimum tenuiflorum (Lamiaceae) and prediction of biological activity of its metabolites. Phcog. Res. 7, 156. 10.4103/0974-8490.151457 25829789 PMC4357966

[B48] Pari L. Latha M. (2002). Effect of Cassia auriculata flowers on blood sugar levels, serum and tissue lipids in streptozotocin diabetic rats. Singapore Med. J. 43, 617–621. 12693765

[B49] Parker S. Moore P. Johnson L. Poitout V. (2003). Palmitate potentiation of glucose-induced insulin release: a study using 2-bromopalmitate. Metabolism 52, 1367–1371. 10.1016/s0026-0495(03)00279-8 14564691

[B50] Patel D. Prasad S. K. Kumar R. Hemalatha S. (2012). An overview on antidiabetic medicinal plants having insulin mimetic property. Asian Pac. J. Trop. Biomed. 2, 320–330. 10.1016/S2221-1691(12)60032-X 23569923 PMC3609288

[B51] Peiris L. D. Dhanushka M. Jayathilake T. (2015). Evaluation of aqueous leaf extract of Cardiospermum halicacabum (L.) on fertility of male rats. Biomed. Res. Int. 2015, 175726. 10.1155/2015/175726 26064883 PMC4439466

[B52] Preethi K. C. Kuttan R. (2009). Hepato and reno protective action of Calendula officinalis L. flower extract. Indian J. Exp. Biol. 47 (3), 163–168. 19405380

[B53] Rajasekaran S. Sivagnanam K. Ravi K. Subramanian S. (2004). Hypoglycemic effect of Aloe vera gel on streptozotocin-induced diabetes in experimental rats. J. Med. Food 7, 61–66. 10.1089/109662004322984725 15117555

[B54] Ramkumar K. M. Vanitha P. Uma C. Suganya N. Bhakkiyalakshmi E. Sujatha J. (2011). Antidiabetic activity of alcoholic stem extract of Gymnema montanum in streptozotocin-induced diabetic rats. Food Chem. Toxicol. 49, 3390–3394. 10.1016/j.fct.2011.09.027 21978819

[B55] Reitman S. Frankel S. (1957). A colorimetric method for the determination of serum glutamic oxalacetic and glutamic pyruvic transaminases. Am. J. Clin. Pathol. 28, 56–63. 10.1093/ajcp/28.1.56 13458125

[B56] Roeschlau P. Bernt P. Gruber W. (1974). Enzymatic determination of total cholesterol in serum. Clin. Chemis. 20 (6), 724–725. 10.1093/clinchem/20.6.724 4440114

[B57] Sasaki M. (1966). A new ultramicro method for the determination of serum alkaline phosphatase. Use of Berthelot’s reaction for the estimation of phenol released by enzymatic activity. Igaku Seibutsugaku 70, 208–214. 5949458

[B58] Sezik E. Aslan M. Yesilada E. Ito S. (2005). Hypoglycaemic activity of Gentiana olivieri and isolation of the active constituent through bioassay-directed fractionation techniques. Life Sci. 76, 1223–1238. 10.1016/j.lfs.2004.07.024 15642593

[B59] Shah N. A. Khan M. R. Ahmad B. Noureen F. Rashid U. Ali Khan R. (2013). Investigation on flavonoid composition and anti free radical potential of Sida cordata. BMC Complement. Altern. Med. 13, 276. 10.1186/1472-6882-13-276 24148097 PMC3874743

[B60] Sharma B. Salunke R. Balomajumder C. Daniel S. Roy P. (2010). Anti-diabetic potential of alkaloid rich fraction from Capparis decidua on diabetic mice. J. Ethnopharmacol. 127, 457–462. 10.1016/j.jep.2009.10.013 19837152

[B61] Sharma U. K. Kumar R. Gupta A. Ganguly R. Singh A. K. Ojha A. K. (2019). Ameliorating efficacy of eugenol against metanil yellow induced toxicity in albino Wistar rats. Food Chem. Toxicol. 126, 34–40. 10.1016/j.fct.2019.01.032 30738991

[B62] Shinde U. Phadke A. Nair A. Mungantiwar A. Dikshit V. Saraf M. N. (1999). Membrane stabilizing activity—a possible mechanism of action for the anti-inflammatory activity of Cedrus deodara wood oil. Fitoterapia 70, 251–257. 10.1016/s0367-326x(99)00030-1

[B63] Shukla S. Mehta A. Mehta P. Bajpai V. (2011). Evaluation of comparative antidiabetic effects of ethanolic extracts of Caesalpinia bouncucella and Stevia rebaudiana in normal and alloxan-induced experimental rats. Rom. Biotechnol. Lett. 16, 6187–6199.

[B64] Stanely P. Prince M. Menon V. P. (2000). Hypoglycaemic and other related actions of Tinospora cordifolia roots in alloxan-induced diabetic rats. J. Ethnopharmacol. 70, 9–15. 10.1016/s0378-8741(99)00136-1 10720784

[B65] Su C. H. Hsu C. H. Ng L. T. (2013). Inhibitory potential of fatty acids on key enzymes related to type 2 diabetes. Biofactors 39, 415–421. 10.1002/biof.1082 23355366

[B67] Swamy M. K. Sinniah U. R. Akhtar M. (2015). *In vitro* pharmacological activities and GC-MS analysis of different solvent extracts of Lantana camara leaves collected from tropical region of Malaysia. Evid. Based Complement. Alternat Med. 2015, 506413. 10.1155/2015/506413 26783409 PMC4689920

[B68] Szkudelski T. (2001). The mechanism of alloxan and streptozotocin action in B cells of the rat pancreas. Physiol. Res. 50, 537–546. 11829314

[B69] Tyagi T. Agarwal M. (2017). Phytochemical screening and GC-MS analysis of bioactive constituents in the ethanolic extract of *Pistia stratiotes* L. and *Eichhornia crassipes* (Mart.) solms. J. Pharmacogn. Phytochem. 6, 195–206.

[B70] Tzeng T.-F. Liou S.-S. Chang C. J. Liu I.-M. (2014). The ethanol extract of *Lonicera japonica* (Japanese honeysuckle) attenuates diabetic nephropathy by inhibiting p-38 MAPK activity in streptozotocin-induced diabetic rats. Planta Med. 80, 121–129. 10.1055/s-0033-1360196 24431014

[B71] Vadivel E. Gopalakrishnan S. (2011). GC-MS analysis of some bioactive constituents of Mussaenda frondosa Linn. Inter. J. Pharm. Bio Sci. 2, 313–320.

[B72] van de Venter M. Roux S. Bungu L. C. Louw J. Crouch N. R. Grace O. M. (2008). Antidiabetic screening and scoring of 11 plants traditionally used in South Africa. J. Ethnopharmacol. 119, 81–86. 10.1016/j.jep.2008.05.031 18588966

[B73] Wu K. K. Huan Y. (2008). Streptozotocin-induced diabetic models in mice and rats. Curr. Protoc. Pharmacol. 5, 1–5. 10.1002/0471141755.ph0547s40 22294227

[B74] Yehya A. H. Asif M. Kaur G. Hassan L. E. Al-Suede F. S. Majid A. M. A. (2019). Toxicological studies of Orthosiphon stamineus (Misai Kucing) standardized ethanol extract in combination with gemcitabine in athymic nude mice model. J. Adv. Res. 15, 59–68. 10.1016/j.jare.2018.05.006 30581613 PMC6300433

